# Comment on “Comparison of analgesic efficacy between rectus sheath blockade, intrathecal morphine with bupivacaine, and intravenous patient-controlled analgesia in patients undergoing robot-assisted laparoscopic prostatectomy: a prospective, observational clinical study”

**DOI:** 10.1186/s12871-021-01304-x

**Published:** 2021-03-17

**Authors:** Lukas M. Löffel, Dominique Engel, Patrick Y. Wuethrich

**Affiliations:** Department of Anaesthesiology and Pain Medicine, Inselspital, Bern University Hospital, University of Bern, Bern, Switzerland

Dear Editor.

We read with great interest the article “Comparison of analgesic efficacy between rectus sheath blockade, intrathecal morphine with bupivacaine, and intravenous patient-controlled analgesia in patients undergoing robot-assisted laparoscopic prostatectomy: a prospective, observational clinical study” by Shim et al. [1] Yet we think that there are relevant methodological issues and therefore we raise concerns about the potential for generalization of the results for other centers.

The observational nature of the trial with patients being able to choose their analgesic regimen themselves poses a significant risk of bias. The patients opting for intrathecal analgesia may be different in their general perception of pain and satisfaction compared to the other groups. According to the flowchart, only 103 patients had to be evaluated for inclusion, of which 13 dropped out because of fulfilling the exclusion criteria. In our experience, it seems unlikely that the remaining 90 participants could be distributed exactly equal to three groups of 30 the trial aimed for, even though they were reported to be able to choose themselves unrestrictedly. The authors state that there were no adverse events such as respiratory depression, which is a well known complication of intrathecal morphine [2]. This occurs with a delay of up to 24 h postoperatively. How were patients monitored for this complication? Furthermore, it is not clear how the sample size analysis was performed. Shim et al. state that it was based on drug consumption of 20 patients on postoperative day 1. There is uncertainty how these patients were identified. Last but not least, the reviewers of this paper also raised concerns that the study is not comprehensive and that the conclusions cannot be sufficiently supported [3].

With our ongoing three-arm randomized controlled trial “Impact of Perioperative Analgesia in Prostatectomy Patients on Early Quality of Recovery” (“SPITALIDO”, registered on clinicaltrials.gov on August 7, 2018 with the identifier NCT03618693) we hope to omit these methodological issues and to provide further data for the studied patient population [4].

## Data Availability

Not applicable.
